# All Microbiological Aspects of SARS-CoV-2 Virus

**DOI:** 10.5152/eurasianjmed.2022.22315

**Published:** 2022-12-01

**Authors:** Murat Karamese

**Affiliations:** 1 Faculty of Medicine, Department of Medical Microbiology, Kafkas University, Kars, Turkey

**Keywords:** SARS-CoV-2, COVID-19, ACE-2, diagnosis, epidemiology

## Abstract

The COVID-19 disease, caused by SARS-CoV-2 virus, which was first seen in Wuhan (China) on December 31, 2019, rapidly spread to cities, countries, and continents and was noted in history as the first pandemic caused by coronaviruses. According to the World Health Organization reports, more than 645 million confirmed SARS-CoV-2-positive cases and more than 6.5 million confirmed deaths were noted all over the world during the pandemic (between December 2020 and December 2022). Although SARS-CoV-2 is a virus belonging to the coronavirus family, our knowledge of the pathogenesis and immune response of SARS-CoV-2 is still limited. Approximately 10 years (2012) after the Middle East Respiratory Syndrome (MERS-CoV) (nearly 2200 confirmed cases and 791 confirmed deaths) and 20 years (2002-2004) after the SARS-CoV epidemic (29 different countries, nearly 8000 confirmed cases, and 774 confirmed deaths), the current COVID-19 pandemic is a reminder of how new pathogens can emerge and spread rapidly, eventually causing serious public health problems. Further research is needed to establish animal models for SARS-CoV-2 to investigate replication, transmission dynamics, and pathogenesis in humans in order to develop effective antiviral treatments and vaccines.

**Main Points**

- The COVID-19 disease, which was first seen in Wuhan (China) on December 31, 2019, rapidly spread to cities, countries, and continents and was noted in history as the first pandemic caused by coronaviruses.
- Approximately 10 years (2012) after the MERS-CoV (nearly 2200 confirmed cases and 791 confirmed deaths) and 20 years (2002-2004) after the SARS-CoV epidemic (29 different countries, nearly 8000 confirmed cases and 774 confirmed deaths), the current COVID-19 pandemic is a reminder of how new pathogens can emerge and spread rapidly, eventually causing serious public health crises.
- It is quite possible that new variants will emerge over time due to various numbers of mutations in coronaviruses. For this reason, detailed further studies on the biology, pathogenesis, epidemiology, immunology, and other aspects of coronaviruses are needed.

## Introduction

COVID-19 disease, caused by the SARS-CoV-2 virus, which is first seen in Wuhan (China) on December 31, 2019, rapidly spread to cities, countries, and continents and was noted in history as the first pandemic caused by coronaviruses.^1^ According to the World Health Organization (WHO) reports, more than 645 million confirmed SARS-CoV-2-positive cases and more than 6.5 million confirmed death are noted all over the world during the pandemic (between December 2020 and December 2022).^2^

The aim of this review is to give more information about all aspects of SARS-CoV-2 for better understanding of the COVID-19 pandemic. Since effective treatment against SARS-CoV-2 has not yet been developed, the potential entry routes of the virus into the body and the mechanisms of action of COVID-19 are of great importance in terms of preventing infection. A clear understanding of the pathophysiology of COVID-19 and its mechanisms of action on the immune system is of vital importance both for the symptomatic treatment of the disease and for the development of possible treatment mechanisms.

## Coronaviruses and Severe Acute Respiratory Syndrome-Coronavirus-2

**Figure 1.**
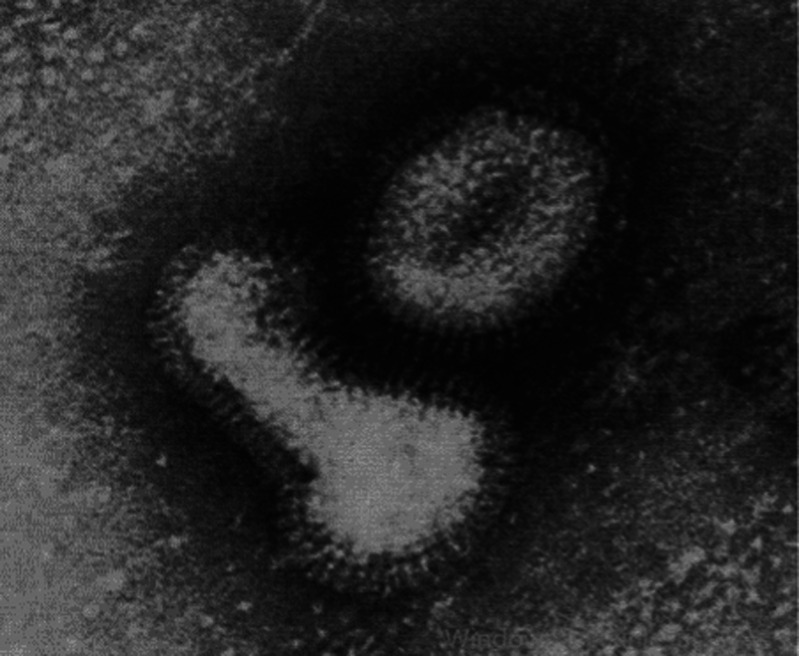
The electron microscopic micrograph of SARS-CoV-2.^4^

The history of human coronaviruses started with the virus called B184, which was first isolated from children with upper respiratory tract infections by Tyrell and Boyne in the early 1960s. At the same time, Hamre and Procknow identified a new virus isolated from the samples of Medical Faculty students who had a cold, by hemagglutination test. The viruses described by Hamre and the B184 virus were named 229E. Towards the end of the 1960s, it was found that this new virus group was not related to the known myxo/paramyxoviruses and their electron microscopic appearance was similar to the viruses that cause bronchiolitis in chickens. This virus group, which was determined to have “crown-like” protrusions on its surface with electron microscope images, was named coronavirus (crowned virus)^3^ (Figure 1).^4^

**Figure 2.**
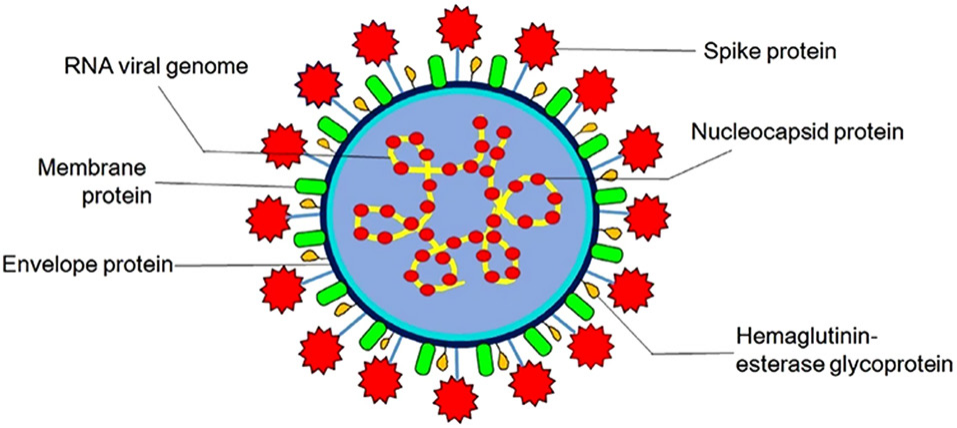
The structural proteins of SARS-CoV-2 virus.^8^

Coronaviruses are single-stranded, positive-polarized, non-segmented, enveloped RNA viruses. Because of their positive-polarized RNA structure, they do not contain the RNA-dependent RNA polymerase enzyme; however, they have the ability to encode this enzyme in their genome. They have the largest genome among all RNA virus, with a length of 27-32 kb. It is well known that mutation rates are much higher in the replication of RNA viruses than in DNA viruses. These mutations can give the virus a new ability to infect new cell types and new species.^5,6^ The coronavirus generally has 4 or 5 major structural proteins: nucleocapsid (N), envelope (envelope; E), membrane (M), hemagglutinin-esterase glycoprotein (HE), and spike (S)^7^ (Figure 2).^8^

In coronaviruses, there are host-originated membranes that are surrounded by glycoprotein spikes and these membranes surround the genome encased in the nucleocapsid. Viral RNA replication occurs through a unique mechanism. During this replication, RNA polymerase binds to a leader sequence in the host cytoplasm and produces a nested set of mRNA molecules with common ends.^6^

Some human coronaviruses (HCoV-229E, HCoV-NL63, and SARS CoV) have 4 structural genes encoding the S, M, N, and E proteins, respectively; however, the additional fifth structural gene encoding the HE protein is available only in the genomes of HCoV-OC43 and HCoV-HKU1. Major proteins of SARS-CoV-2 are mentioned in detail under the title “SARS-CoV-2 Genome;” however, to summarize briefly, the S protein extends through the viral envelope and shapes the characteristic spikes in the coronavirus crown. It has an important role in mediating receptor binding with the host cell membrane. The other major protein, M protein, has a short N-terminal domain that protrudes on the outer surface of the envelope. The M protein is crucial for viral assembly. The N protein has different roles in genome. It is responsible for the formation of nucleocapsid and the regulation of viral RNA synthesis. N and M proteins are in contact during the budding step. On the other hand, the fifth protein, HE, is only found in beta-coronaviruses, HCoV-OC43, and HKU1. The hemagglutinin binds to the neuraminic acid on the host cell surface and probably allows initial adsorption of the virus to the membrane. Acetyl groups are cleaved from neuraminic acid by esterase.^9^

The phylogeny analysis and whole-genome sequencing showed that SARS-CoV-2, the viral agent of COVID-19 disease, is also a beta-coronavirus like the former epidemic viral agent (SARS-CoV) but in a different clade. The similarity between SARS-CoV and SARS-CoV-2 starts with the structure of receptor-binding gene region, and it has been detected that both viruses use the same receptor, angiotensin-converting enzyme 2 (ACE2), for cell entry. MERS-CoV, another beta-coronavirus, seems to be more distantly related in this respect. There is a quite similarity in terms of RNA sequences of those 2 bat coronaviruses, and this suggests that bats are the primary source. It is not known whether the SARS-CoV-2 virus is transmitted directly from bats or through another mechanism (e.g., through an intermediate host).^10^ One of the first phylogenetic analysis studies performed in China with 103 SARS-CoV-2 strains reported that 2 different SARS-CoV-2 strains, designated as type L (70% of strains) and type S (30%), were identified. Type L was predominant in the first days of the epidemic in China.

## Severe Acute Respiratory Syndrome-Coronavirus-2 Genome

**Figure 3.**
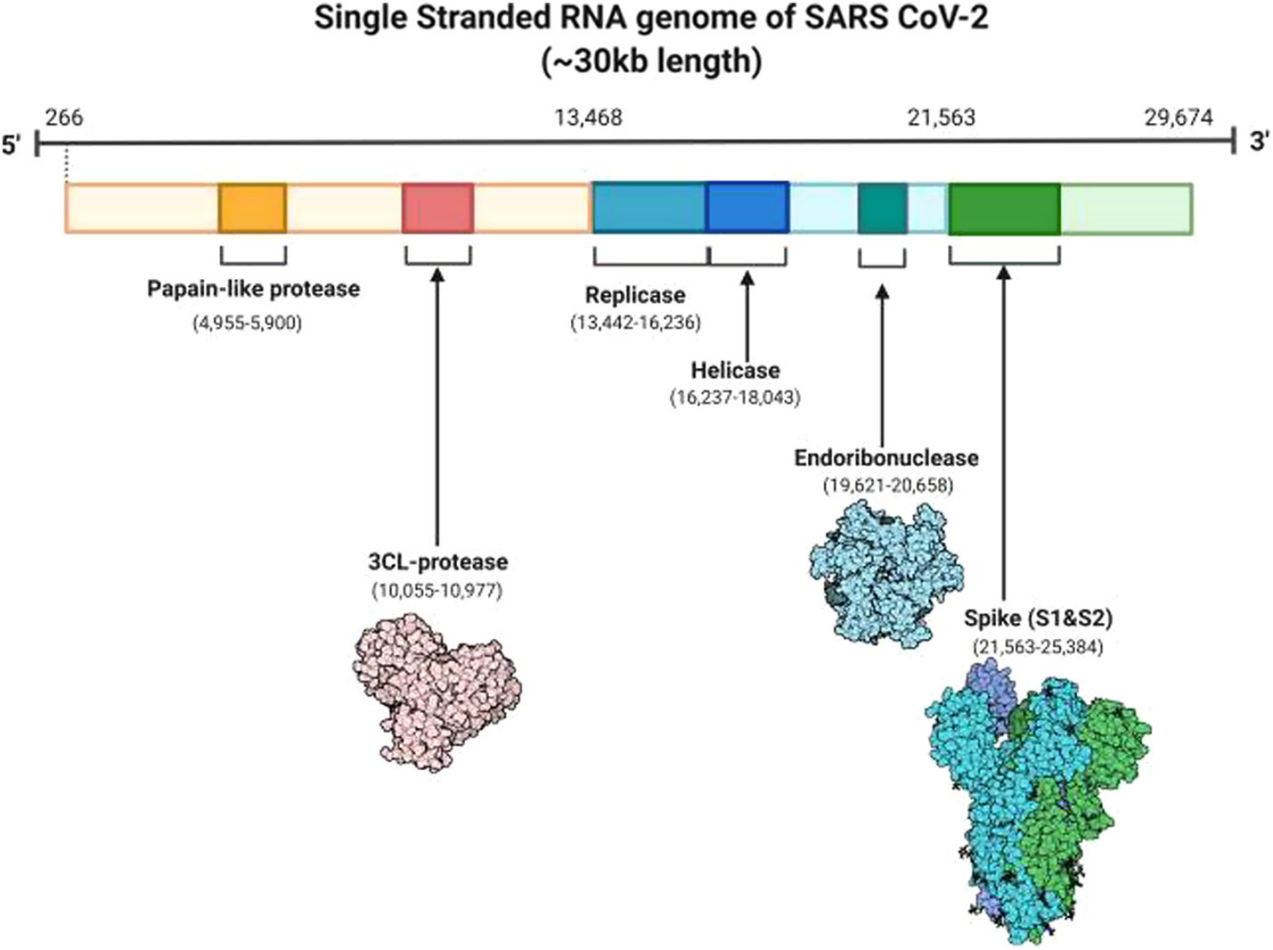
The genomic structure of SARS-CoV-2.^16^

Although SARS CoV-2 is an enveloped virus, it has a single-stranded, positive-polarized, and unsegmented RNA genome.^11,12^ Its genome is approximately 30 000 bp long and Guanine-Cytosine (GC) content is 38%.^13^ There are 2 non-protein-coding regions in the 5’ and 3’ regions of the viral genome. According to the data obtained from genome sequence analysis, it has been established that SARS-CoV-2 genome contains 14 open reading frames (ORFs) encoding 27 proteins. It has 4 structural proteins, namely, envelope (E), membrane (M), nucleocapsid (N), and spike (S), which are encoded by different ORFs.^14^ Each major protein has specific roles in the genome structure of SARS-CoV-2 in addition to involvement in the replication cycle^15^ (Figure 3).^16^

The N protein is about 50-60 kDa, is part of the nucleocapsid, and is the only protein that binds to the RNA genome. This protein consists of 2 separate domains, the N-terminal domain and the C-terminal domain. It is thought that both domains should contribute to optimal RNA binding. It protects the viral genome from outside host cells. It also plays a role in viral assembly and budding, resulting in virion formation.^17,18^

The M protein is about 25-30 kDa and has 3 transmembrane domains. The M protein is the most abundant structural protein and forms the shape of the viral envelope.^19^ Binding of the M protein to N protein stabilizes the nucleocapsid and the interior of the virions and helps the viral maturation process. It also gives the virion its shape.^20^ In viral organization, it is exactly required that S protein interacts with M protein for attachment to the endoplasmic reticulum-Golgi-mediated compartment (ERGIC)/Golgi complex and its incorporation into new virions.^21^ Another interaction is necessary between M protein and E protein to produce and release virus-like particles (VLPs).^22^

The E protein is about 8-12 kDa, and the smallest among all SARS-CoV-2 major proteins. It is also a transmembrane protein with an N-terminal and a C-terminal endodomains. According to the current data, E protein is mostly expressed inside the infected cells and plays a crucial role in virus assembly, viral maturation, and budding.^23,24^

The S protein is about 150 kDa and helps in the attachment of virus to the host cell surface receptors resulting in fusion and then viral entry.^25-27^ This protein is also strongly N-linked glycosylated. Generally, most of the coronaviruses’ S protein is cleaved by host protease into S1, is responsible for receptor binding, and S2, is responsible for cell membrane fusion.^28^

On the other hand, ORF-1 is the first ORF, corresponds to nearly 2/3 the length of total viral RNA, and has 2 reading frames (ORF1a and ORF1ab). Open reading frames-1ab codes 16 different non-structural proteins and one of them is RNA-polymerase. Others have role in suppressing innate immunity and blocking the translation of host cell RNA. In contrast, other ORFs such as ORF3a, ORF6, ORF7a, ORF7b, ORF8, and ORF10 code at least 6 different accessory proteins.^29,30^ All mentioned accessory proteins and major structural proteins are located in the 3’-terminus of the SARS-CoV-2 genome.^31^

## Phylogenetic Analysis and Molecular Differences

Phylogenetic analyses confirm that the virus causing COVID-19 pandemic may have genetically different types, since viruses in the Coronaviridae family, of which SARS CoV-2 is a member, have the second longest genome among all RNA viruses and have a high mutation rate.^15^ In the initial stages of the COVID-19 pandemic, a study by Tang et al^10^ determined that there are 2 types of SARS CoV-2, called S and L. When the whole-genome sequences of 103 SARS CoV-2 viruses were analyzed by Tang et al. (2020)^10^, it was seen that the samples were located in the phylogenetic tree in accordance with the nucleotide (nt) differences in positions 8782 and 28144 compared to the sequence of the reference Wuhan-Hu-1 isolate. Then, it has been determined by Global Initiative on Sharing All Influenza Data (GISAID) database experts that SARS CoV-2 has V, G, GH, and GR types in addition to S and L types, and it has totally been divided into 6 types.^32^

**Figure 4.**
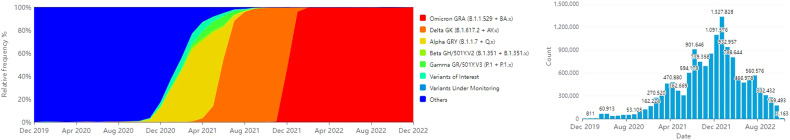
The distribution of SARS-CoV-2 variants during the pandemic.

In addition to the S protein, one of the structural proteins of SARS-CoV-2, the presence of mutations that cause aminoacid (aa) change in N and M proteins has been detected in many scientific studies.^33,34^ According to those mutations, SARS-CoV-2 variants are named omicron, delta, alpha, beta, gamma, etc. The emergence of new SARS CoV-2 types due to mutations occurring over time and which type is more circulating worldwide are better understood in the phylogenetic analysis prepared by GISAID experts using 14,164, and 794 SARS CoV-2 sequence data obtained between December 2019 and December 2022.^32^ The distribution of SARS-CoV-2 variants from the beginning of pandemic untill now is given in Figure 4.

## Replication

There are some key steps in the replication cycle of the SARS-CoV-2 virus infection in the host cell. These steps can be ordered as follows: (i) viral adhesion, (ii) cell entry, (iii) transcription of viral replicase, (iv) genomic transcription and replication, (v) translation of structural proteins, and (vi) virion assembly and release.^5,35^

The viral replication of coronaviruses takes place in the cytoplasm of the host cell. According to the current data, receptors have been identified for some coronaviruses. Many alpha-coronaviruses bind to aminopeptidase N as a receptor. On the other hand, MERS-CoV binds to dipeptidyl-peptidase 4, while HCoV-NL63, SARS-CoV, and SARS-CoV-2 bind to different regions of ACE2, which is located in the vascular endothelial cells, small intestine, and respiratory system epithelium. This attachment (ACE-2 vs. S protein) is necessary for the virus to enter the cell.^36^

**Figure 5.**
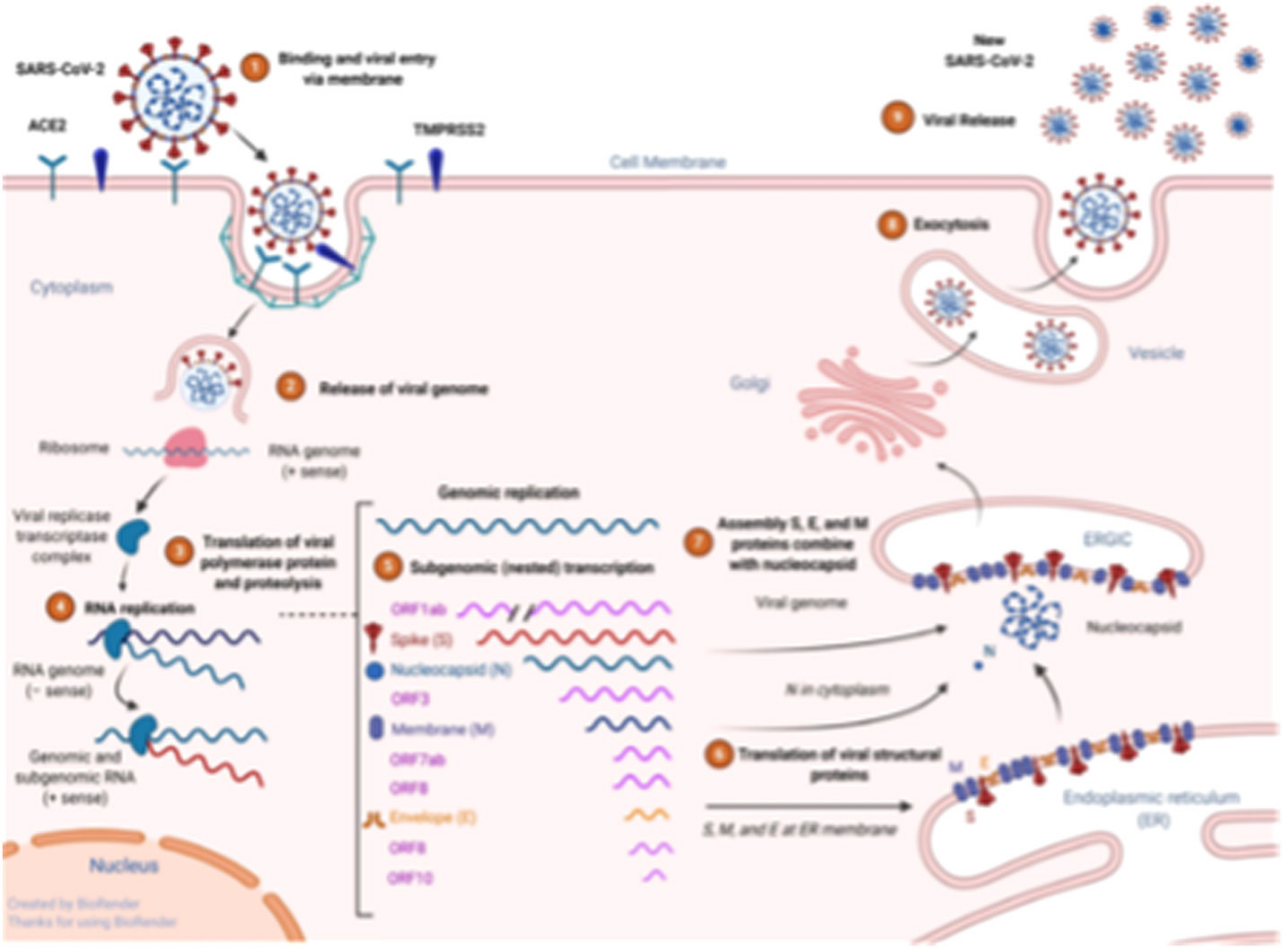
The life cycle of SARS-CoV-2 virus.^46^

Following the receptor binding, the virus must then reach the host cell cytoplasm. For this, fusion of viral and cellular envelopes takes place after S protein cleavage, usually with the help of proteases such as acid-dependent cellular transmembrane serine protease 2 (TMPRRS2). After the virus enters the cytoplasm, the RNA genome is released. The next step in the virus’s replication cycle is the translation of the replicase gene (first gene) from genomic RNA. The replicase gene is 2 large ORFs expressing 2 coterminal polyproteins pp1a and pp1b located at the 5’ end of genomic RNA. It encodes the ORF1a and ORF1b gene regions. After the synthesis of 2 polyproteins, it is converted into 16 non-structural proteins (nsp1-nsp16) with the help of viral proteases.^6,37,38^ Of these proteins, especially nsp3 has an important role in the virion structure, replication, and transcription of coronaviruses. In addition, many nsps form the replicase–transcriptase complex to provide an appropriate condition for RNA synthesis.^39,40^ Consequently, nsps are responsible for RNA replication and transcription of subgenomic RNAs. After translation and maturation of viral replicase complexes, viral RNA is synthesized. At this stage, both genomic and sub-genomic RNAs are synthesized. Sub-genomic RNAs serve structural and accessory genes in replicase polyproteins just like mRNAs.^18^ Regions of 2-7 genes encoding viral structural proteins (S, E, M, N) and accessory proteins are translated from sub-genomic mRNAs. The newly synthesized structural proteins, together with the N protein, are localized in the ER-to-Golgi intermediate compartment (ERGIC) in association with the viral genome.^41,42^ In this complex, viral proteins and genome form the nucleocapsid together with the N protein and are transported in the form of vesicles and released from the cell by budding. Although the N protein is required for coronavirus replication, its role in this process is unknown. However, many studies have reported that the interaction of this protein with nsp3 plays a critical role in early viral replication during infection.^6,43-45^ All steps of viral replication are summarized in Figure 5.^46^

## Epidemiology

**Figure 6.**
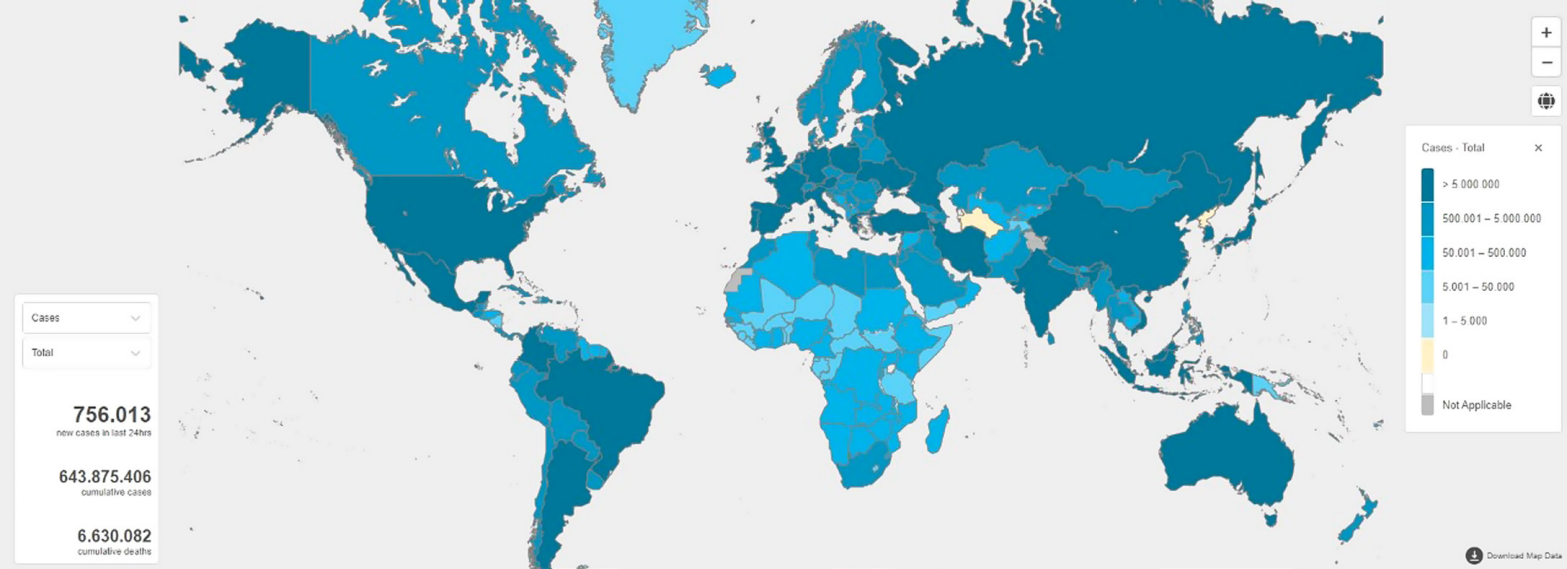
Total number of COVID-19 confirmed cases and deaths during the pandemic.^49^

Coronaviruses are known as important human and animal pathogens. In the last days of 2019, some pneumonia cases in the Huanan Seafood Market in Wuhan, China, were associated with a new coronavirus epidemic, initially named 2019-nCoV and was named SARS-CoV-2 in the later stages of the epidemic. In January, the first imported case was seen in Thailand. Then, many countries, especially Japan, South Korea, and America, started to report imported cases. On January 30, WHO declared the Coronavirus an International Health Emergency and on February 11, WHO announced that the disease caused by the new coronavirus would be named “coronavirus disease-2019 (COVID-19)”.^6,47,48^ At present, there are 647 972 911 confirmed cases, and 6 642 832 confirmed death recorded by WHO^2^ (Figure 6).^49^

However, SARS-CoV-2 positivity has been reported on all continents except Antarctica. Since the beginning of the epidemic, most cases and deaths have been seen in the United States of America. Our country is in the 11th in terms of confirmed cases and deaths duringCOVID-19 pandemics^49^ (Table 1).

## Transmission

**Figure 7.**
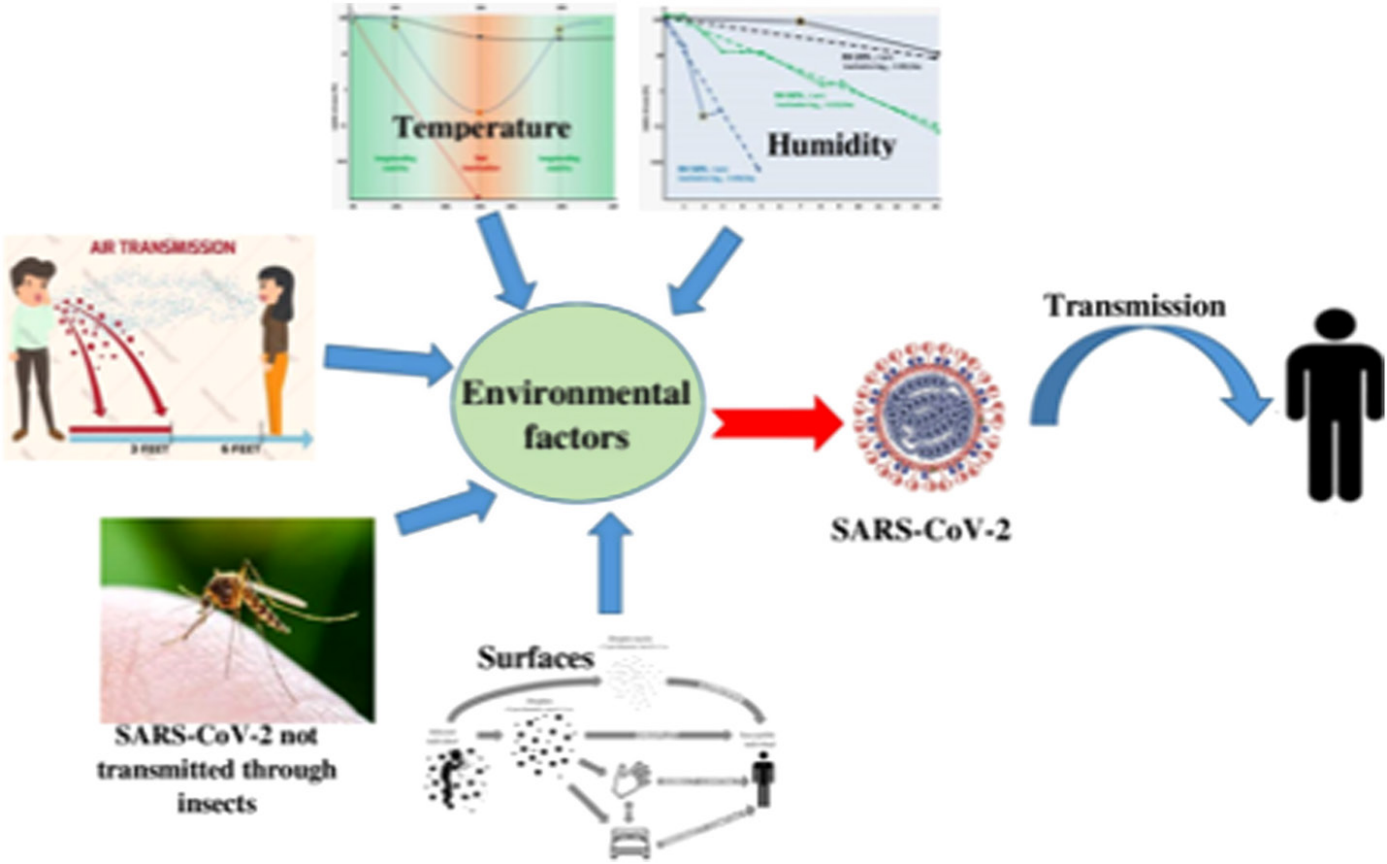
Environmental factors and transmission of SARS-CoV-2 virus.^54^

It has been reported that coronavirus, which was first detected in 1962 in respiratory system of a human, has a wide host range and infects many animal species such as camel, cattle, cat, dog, and bat.^7^ As like other respiratory viruses, SARS-CoV-2 transmission comes out with high efficacy and infectivity mainly through the respiratory route. The virus is transmitted from person to person mainly by droplets and at a distance of less than 2 m.^50^ It remains infectious in droplets (less than 5 µm in diameter) intact and can be suspended in the air for up to 3 hours. Although the duration of the contagiousness of the disease is not exactly known, it is thought to start 1-2 days before the onset of symptoms and continue until the disappearance of the symptoms. Since coronaviruses are enveloped viruses, their resistance to the external environment is low. Durability to the external environment depends on some factors such as the humidity of the environment, temperature, and texture of the surface. In a study, it was reported that it remained on metal, glass, and plastic surfaces for up to 9 days. Another study reported that the virus continued its activity for up to 4 hours on copper surfaces, up to 24 hours on cardboard, and up to 72 hours on plastic and stainless steel. However, it is generally accepted that the virus loses its activity on inanimate surfaces within a few hours.^51-53^ Environmental factors and transmission of SARS-CoV-2 virus are summarized in Figure 7.^54^

On the other hand, the presence of the virus was detected in different human samples including whole blood, serum, urine, and fecal samples.^55^ A study about the fecal–oral transmission of SARS-CoV-2 virus reported that viral RNA was detected in the stool samples of COVID-19 patients. One of the other important human sample is saliva in the detection of SARS-CoV-2 virus. Karamese et al^56^ performed a study on 28 asymptomatic and 25 mildly symptomatic confirmed COVID-19 patients to evaluate the diagnostic value of saliva samples and reported that saliva can be considered as a reliable and less resource-intensive alternative specimen to nasopharyngeal specimens for screening asymptomatic SARS-CoV-2 infections with the percentage of 90.56%. This result also supports the proof of the presence of ACE2 receptors in epithelial cells lining the salivary gland ducts.^57^ In some studies, urine was used to detect the SARS-CoV-2 viral RNA. Although the positivity rate was detected too low (5%-6%) in some studies, it can be said that the virus can be found in urine sample.^58^

In addition, the vertical transmission of SARS-CoV-2 virus is not clearly proven. A study performed on 9 pregnant women with COVID-19 disease reported that no transmission was detected from mothers to newborns and the virus was not detected in breast milk.^59^ However, studies are still being performed to find any clue about the vertical transmission of SARS-CoV-2 virus. Finally, the eyes may be another important route of transmission of virus. Studies are available that show the SARS-CoV-2 virus in ocular swabs.^39^

## Diagnosis

**Figure 8.**
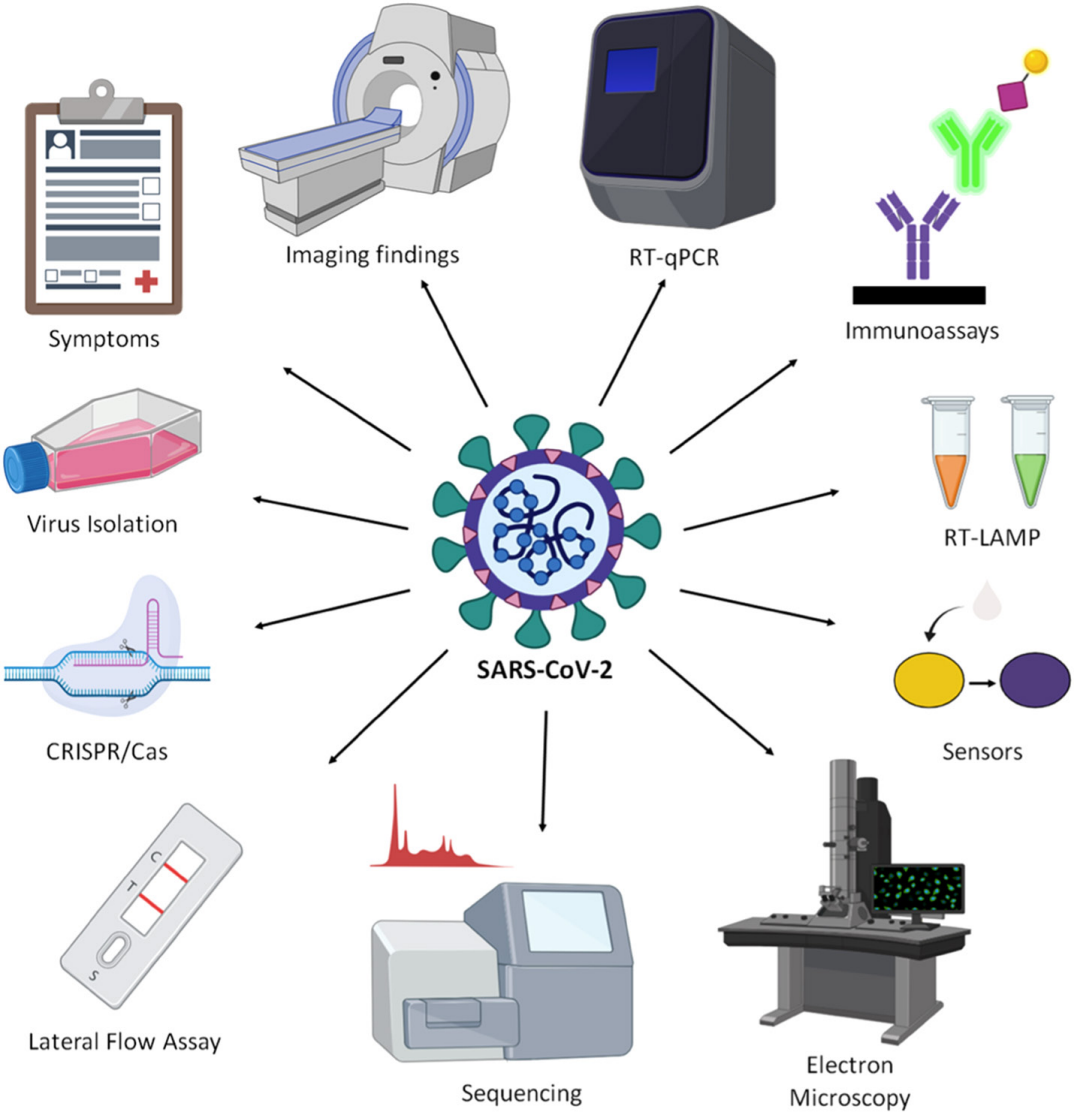
All diagnostic methods of SARS-CoV-2.^61^

The COVID-19 pandemic has become one of the most serious health problems in human history. According to the experience gained in past epidemics and the ongoing COVID-19 pandemic, there are features that an ideal diagnostic test for a rapidly spreading viral pathogen should have accuracy, reliability, and fast results, easy application, and low cost. According to the information obtained to date, the SARS-CoV-2 virus is mainly transmitted to humans through the respiratory tract, first colonizing the nasopharynx and gastrointestinal tract and then causing lung involvement. The tests used in the laboratory diagnosis of SARS-CoV-2 infection can be divided into 2 main groups as molecular methods and immunological (serological) methods. The molecular methods can be used in all terms of disease as a diagnostic test, while antibody detection-based tests provide reliable results from 10 to 14 days of disease course.^60^ A summary of COVID-19 diagnostic approach is given in Figure 8.^61^

Molecular tests are used to identify symptomatic or asymptomatic carriers of SARS-CoV-2. Currently, there are more than 300 different commercial kits for the detection of SARS-CoV-2 viral genome recommended by the WHO.^60^ The main performance criteria for these tests are high sensitivity and low false-negative results. The laboratory method that has been widely used in the molecular diagnosis of COVID-19 since the beginning of the pandemic process is real-time polymerase chain reaction (real-time RT-PCR). Real-time polymerase chain reaction determines the amplified SARS-CoV-2 genome in some human samples including sputum, nasopharyngeal aspirate or swabs, bronchoalveolar lavage fluid, and lower respiratory tract aspirates. There are different RNA gene targets used by the manufacturers; however, most of the RNA gene targets contain at least one of the following structural proteins; envelope (E), nucleocapsid (N), spike protein (S), RNA-dependent RNA polymerase (RdRp), and ORF1.^62^ Basically, RT-PCR method is based on amplification of specific SARS-CoV-2 genomic sequences or sequences. To perform RT-PCR method, viral RNA is first isolated from any kind of human biological samples and then purified. Purified viral RNA is converted into complementary DNA (cDNA) by using reverse transcriptase (RNA-dependent DNA-polymerase enzyme). The cDNA is amplified by PCR in 3 steps as follows; (i) denaturation of cDNA at 95°C, (ii) binding of primers and probe to denatured cDNA strands at 60°C, and (iii) synthesis of RNA copies by DNA polymerase at 72°C. The amplified PCR products follow the same cycle to make multiple copies of RNA.^63^ The probes labeled with different fluorescent dyes (FAM, HEX, ROX, Cy5, etc.), bind to specific gene regions and produce fluorescent signals that increase simultaneously with amplification are detected by the PCR device through specific channels. There is a direct proportionality between the amount of amplification product and the fluorescence intensity. After the RT-PCR process is completed, the amplification curves of the patient samples and controls, which are directly proportional to the fluorescence, are evaluated.^64,65^ When interpreting the test results, if the threshold (Ct) value of the target gene is less than or equal to the cut-off Ct value, the result is interpreted as positive. In general, Ct < 40 for the diagnosis of COVID-19 infection is considered clinically positive.

Molecular tests have both advantages and disadvantages. These tests provide highly sensitive detection of SARS-CoV-2 viral RNA. But when used to diagnose during a pandemic, they are highly complex, expensive, and slow. Moreover, molecular methods require qualified laboratory personnel and equipped laboratories.^66^ Unsuitable sample collection, storage, or transfer may cause false RT-PCR results. On the other hand, the quality of extracted RNA affects the experiment’s results. In PCR experiments, some factors such as degradation of purified RNA and presence of RT-PCR inhibitors, or genomic mutations can cause false-negative results. Furthermore, cross-contamination of samples and/or technical errors during processing of samples can cause false-positive results. Despite the possibility of all these adverse events, real-time RT-PCR is currently the most accurate and sensitive diagnostic method for the earliest and large-scale detection of SARS-CoV-2.^67^ In addition to RT-PCR, loop-mediated isothermal amplification (LAMP) and clustered regularly interspersed short palindromic repeats (CRISPR) methods for the diagnosis of COVID-19 have been developed by some manufacturers to get highly sensitive and specific results in a shorter time.^68^

Sequence analysis is very important in terms of identifying the virus source, understanding the transmission dynamics, identifying changes in the virus genome, and monitoring mutations. Periodic sequence analysis of the samples is recommended, especially in terms of detecting mutations that may occur in the primer and probe-binding sites that may affect the performance of RT-PCR tests. In addition, sequence analysis is recommended to confirm the diagnosis of COVID-19 in a patient whose RT-PCR result is suspiciously positive. However, sequence analysis is not a practical method for routine diagnosis of COVID-19. It is a method that can be applied in well-equipped reference laboratories due to its high cost, experienced personnel, and intensive workload.^66^

Loop-mediated isothermal amplification PCR was first used in 2000. Due to its ease of application, it is becoming an increasingly popular method in the diagnosis of various viruses such as human immunodeficiency virus, Japanese encephalitis virus, Chikungunya virus, human papillomavirus, dengue virus, West Nile virus, mumps virus, SARS, and MERS viruses. It draws attention with its high sensitivity, faster speed, and less cost compared to conventional PCR.^69^ The CRISPR system has been used in the development of molecular diagnostic kits for the detection of dengue virus, human papillomavirus, and Zika virus in human samples. However, the use of CRISPR-Cas/gRNA complexes to detect nucleic acids has become widespread in recent years. For the use of CRISPR-based tests in the diagnosis of COVID-19, the FDA granted emergency use approval on May 6, 2020.^67^

Serological testing is an important tool for surveillance and epidemiological research, such as understanding the transmission dynamics of the virus in the general population. In contrast to direct viral detection methods, such as nucleic acid amplification tests, antibody tests help determine whether the person has been infected before or not even if they have never shown symptoms. Studies have shown that enzyme-linked immunosorbent assay-based immunoglobulin (Ig) M and IgG antibody tests have high specificity in the serological diagnosis of COVID-19. Enzyme-linked immunosorbent assay and chemiluminescence methods have been developed to detect the concentration of antibodies responsible for the coronavirus spike (S) and nucleocapsid (N) proteins. In addition, the receptor-binding domain along with the S protein is a target of interest for detecting the presence of antibodies specific to SARS-CoV-2.^70^

One of the most important steps of primary immune response in COVID-19 disease is the production of specific antibodies by B cells against this SARS-CoV-2 virus. Neutralizing antibodies can be detected in nearly 50% of infected patients and in all infected patients by days 7 and 14, respectively.^71^ A scientific study established a timeline for the detection of immune response antibodies and revealed that SARS-CoV-2-specific antibodies can be detected only after 8 days. In another study, it was stated that both anti-SARS-CoV-2 IgM and IgG levels increased gradually with the stages of infection, and IgM was detected as early as 3 days and IgG as early as 4 days. Additionally, serum IgA is another diagnostic predictor along with IgG and IgM.^72,73^

As a non-specific laboratory detection of SARS-CoV-2, high levels of aspartate aminotransferase, C-reactive protein, and erythrocyte sedimentation rate can be seen in patients. In severe COVID-19 patients, high levels of alanine aminotransferase, C-reactive protein, d-dimer, lactate dehydrogenase, and some cytokines including interleukin (IL)-2, IL-6, IL-10, and tumor necrosis factor (TNF)-alpha were detected. In addition to all those, decreased lymphocytes and leucopenia and increased creatine kinase MB, and procalcitonin were detected in pediatric patients.^28^

## Immunopathogenesis

The genome sequence similarity of SARS-CoV-2 with SARS-CoV is 79%. Therefore, the pathophysiology of COVID-19 is very similar to that of SARS-CoV infection. The first step in COVID-19 is the binding of the virus to the host cell with its receptor, ACE2. This virus especially targets the cells that express the ACE2 receptor such as airway epithelial cells, alveolar epithelial cells, vascular endothelial cells, and macrophages.^74,75^ Angiotensin-converting enzyme 2 is expressed at different levels in almost all organs in the body. High ACE2 expression is also observed in the intestinal epithelium.^76^ Cardiovascular complications in some patients can be explained by the expression of ACE2 in cardiac cells and vascular endothelium. Angiotensin-converting enzyme 2 is also expressed at lower levels in monocytes and macrophages, which may provide an entry mechanism for SARS-CoV-2 into immune cells. As a result, SARS-CoV-2 has been observed to infect immune cells, including monocytes, macrophages, and T cells.

After SARS-CoV-2 binds to the host cell receptor and is taken up by the endosome, it is recognized by endosomal RNA receptors such as toll-like receptors (TLR)-3, TLR-7, retinoic acid-linked gene I (RIG-I), and melanoma differentiation-associated protein 5 (MDA5). Toll-like receptor 3/7 activation usually results in nuclear translocation of transcription factors Nuclear Factor Kappa B (NFκB) and Interferon Regulatory Factor-3 (IRF3), while RIG-1/MDA5 activation results in IRF3 activation. NFκB activation elevates the expression of pro-inflammatory cytokines (IL-1, IL-6, TNF-α) and type I interferons (T1-IFN). SARS-CoV-2 virus increases IL-1-beta expression through pyroptosis. Increased IL-1-beta expression triggers the increase of IL-6, IL-12, IL-17, Interferon gamma (IFN-gamma), Monocyte Chemoattractant Protein-1 (MCP-1), and Interferon-inducible Protein-10 (IP-10) and causes a local inflammation wave.^77-80^

Following this process, macrophages, dendritic cells, epithelial cells, endothelial cells, monocytes, and B cells in tissues phagocytose viral peptides and present them to T cytotoxic or T helper cells, respectively, with HLA I and II molecules. In addition, some cytokines, chemokines, and immune molecules secreted by antigen-presenting cells (APCs) are important in determining the direction of the reaction. The secretion of such cytokines and chemokines triggers the immune cells to migrate from the blood to the infected area. It triggers the T helper-1 cell response. The lymphopenia and elevated neutrophil-to-lymphocyte ratio that is detected in nearly 80% of COVID-19 patients are the results of lung recruitment of immune cells and infiltration of lymphocytes into the the airways.

The adaptive immune response by T and B cells can be detected in blood tissue approximately 7 days after the onset of COVID-19 symptoms. Generally, most patients recover after immune cells clear the infection site; however, an excessive immune response that triggers the secretion of numerous cytokines including IL-7, IL-8, IL-10, granulocyte colony-stimulating factor (G-CSF), IP-10, MCP-1, macrophage inflammatory protein-Ia (MIP-1a), and TNF can be seen in some patients. Then, this finalizes with diffuse lung inflammation. Interleukin-6 levels continue to increase in these patients over time and are relatively higher in especially deceased patients than in survivors.^69^

Studies on SARS-CoV show that multiple viral structural and nonstructural proteins antagonize interferon responses. This antagonistic effect prevents recognition of viral RNA and suppresses many signaling pathways primarily associated with interferon. Antagonizing the interferon response helps viral replication and results in increased release of pyroptose products. Increased inflammatory cell infiltration may mediate lung damage through the hypersecretion of proteases and reactive oxygen species. Additionally, this results in extensive alveolar damage, including desquamation of alveolar cells, hyaline membrane formation, and pulmonary edema.^81,82^

## Conclusion

In conclusion, although it is a virus belonging to the coronavirus family, our knowledge of the pathogenesis and immune response of SARS-CoV-2 is still limited. Approximately 10 years (2012) after the MERS-CoV (nearly 2200 confirmed cases and 791 confirmed deaths) and 20 years (2002-2004) after the SARS-CoV epidemic (29 different countries, nearly 8000 confirmed cases and 774 confirmed deaths), the current COVID-19 pandemic is a reminder of how new pathogens can emerge and spread rapidly, eventually causing serious public health problems. Further detailed studies are needed to investigate the replication, transmission dynamics, and pathogenesis of SARS-CoV-2 virus using animal models in order to develop effective antiviral treatments and vaccines.

## Figures and Tables

**Table 1.** Current Coronavirus Dashboard (December 2019 to December 2022)

No	Country	Total Cases	Total Deaths
0	Global	643 875 406	6 630 082
1	United States of America	98 072 469	1 074 367
2	India	44 674439	530 653
3	France	37 252 086	155 898
4	Germany	36 726 061	158 851
5	Brazil	35 497781	690 577
6	Republic of Korea	27 611 555	30 975
7	Japan	25 687 798	51 062
8	Italy	24 488 080	181 733
9	United Kingdom	24 053 576	197 723
10	Russian Federation	21 650 659	392 506
11	Turkey	16 919 638	101 203
12	Spain	13 614 807	116 108
13	Vietnam	11 519 011	43 178
14	Australia	10 717 637	15 361
15	China	9 862 129	30 717
